# Supplementary material for: Breast cancer genome and transcriptome integration implicates specific mutational signatures with immune cell infiltration
Source: Nat Commun. 2016 Sep 26;7:12910. doi: 10.1038/ncomms12910 (PMC5052682; doi:10.1038/ncomms12910)
Supplement: Supplementary Information — Supplementary Figures 1 - 9 and Supplementary Tables 1 - 3 [file ncomms12910-s1.pdf]

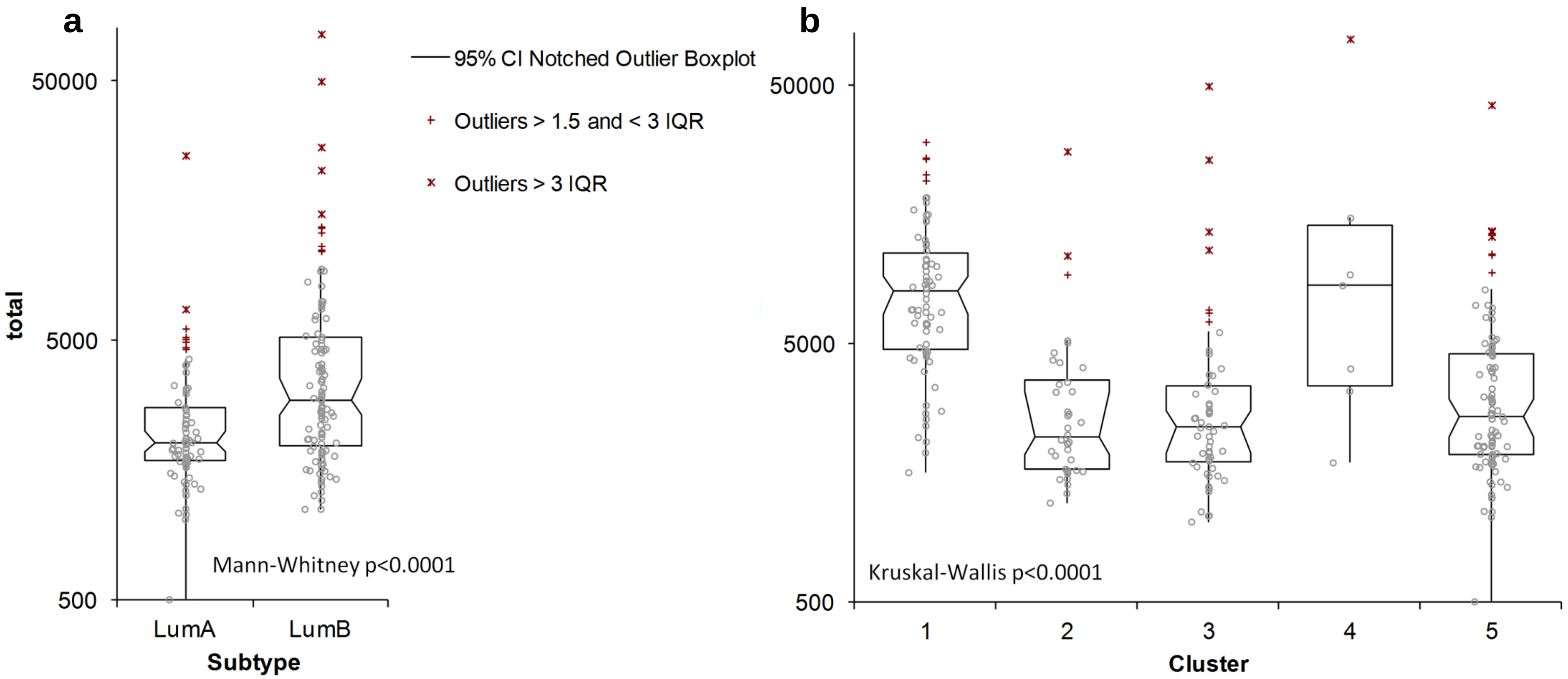

Supplementary figure 1: Number of substitutions between luminal subtypes and clusters of figure 1.

a: number of substitutions in the Luminal A and Luminal B cases, as predicted using the AIMS method. **b**: number of substitutions across the 5 cluster groups from figure 1. IQR is interquartile range.

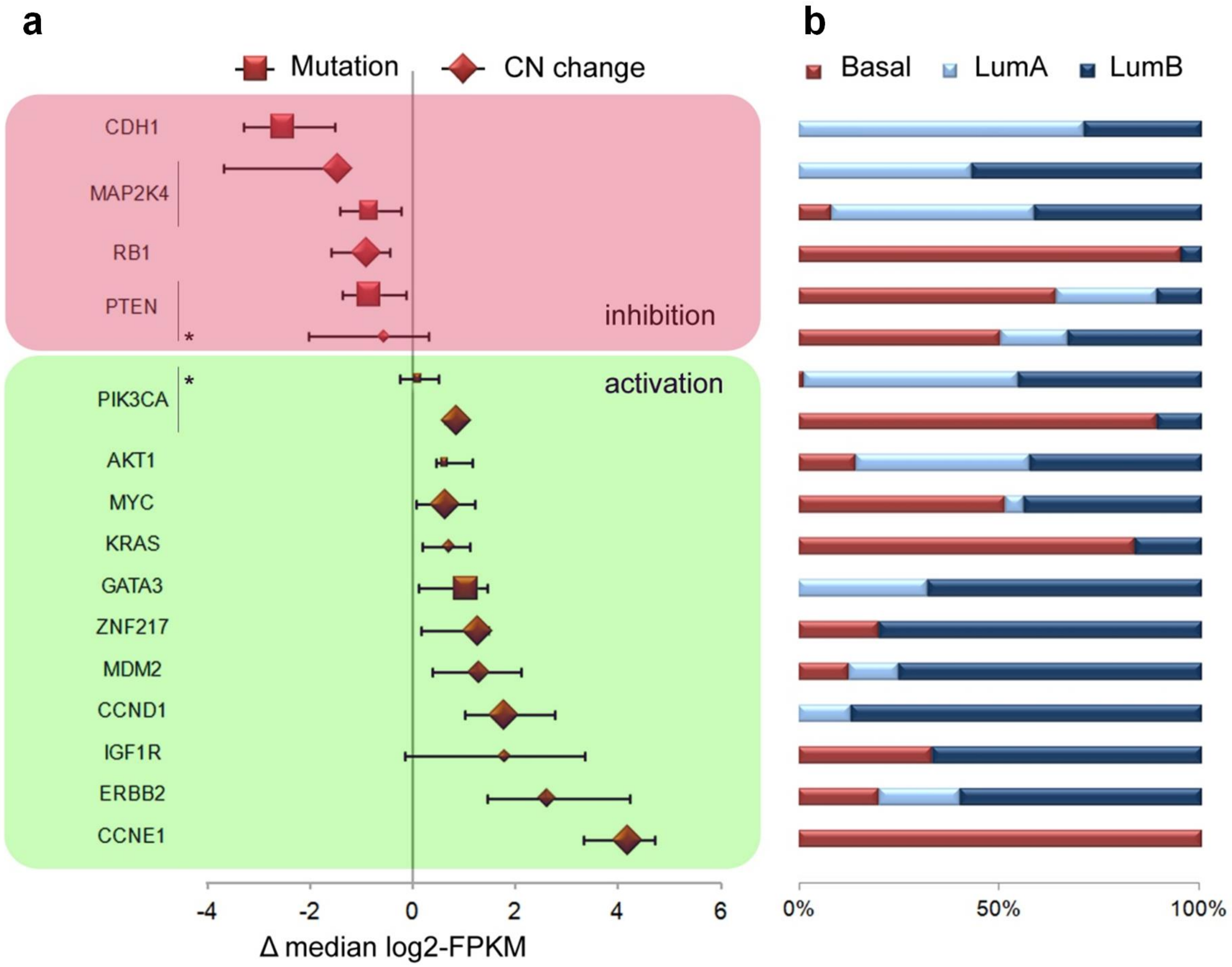

Supplementary figure 2: Expression distribution of breast cancer driver genes.

a shows the difference in FPKM values from the median wild-type expression (set at 0 separately for each gene) in log2 scale. Markers indicate the median and range (first to third quartile) of expression levels in non-wild-type cases. Diamond shape indicates copy number change; deletion for MAP2K4, RB1 and PTEN, and amplification for the other genes. Square markers indicate mutations, which can be substitutions (nonsense, missense, indels, etc) or rearrangements. The size of the marker indicates the Mann-Whitney U-test p-value in 3 categories (big to small size): <0.0001, <0.001 and <0.01. For MAP2K4, PTEN and PIK3CA the Kruskal-Wallis test was used to establish significance, with a post-hoc analysis to check the individual groups; *are groups not significant in this post-hoc analysis. **b** the proportion of affected samples in the basal and luminal subtypes.

a

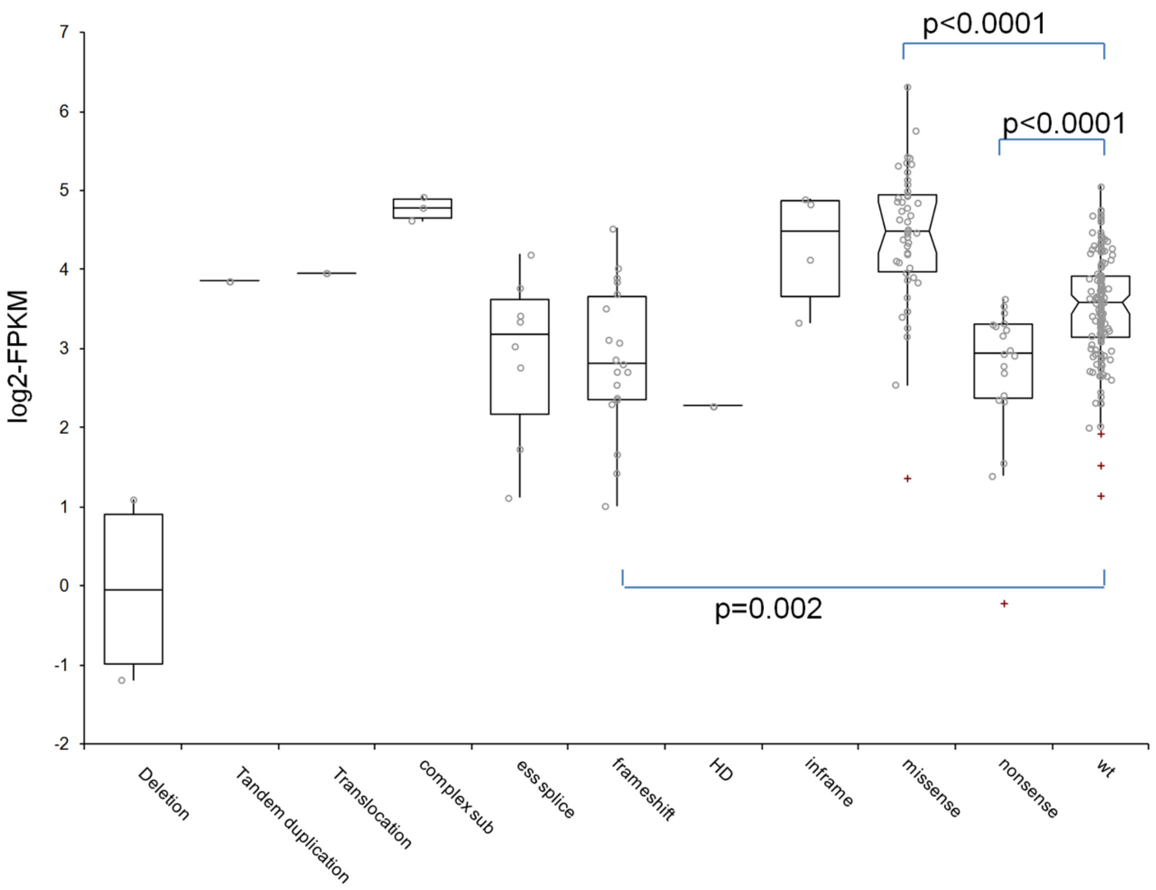

b

**Effect**	TP53_cnnLOH no	TP53_cnnLOH yes
Deletion	3	1
Tandem duplication	2	1
Translocation	1	1
complex sub	2	1
ess splice	15	3
frameshift	25	8
HD	1	0
inframe	5	1
missense	91	9
nonsense	21	11
WT	293	5

Supplementary figure 3: TP53 mutation status versus expression and LOH.
a: Expression (log2-FPKM) of TP53 in the diverse range of TP53 alterations. P-values are derived from the Mann-Whitney test.
b: Copy number neutral LOH status of TP53 versus mutation status. N.B. Numbers differ between left and right panel due to differences in overlap between RNAseq and DNAseq cohort, and copy-number cohort and DNAseq cohort.

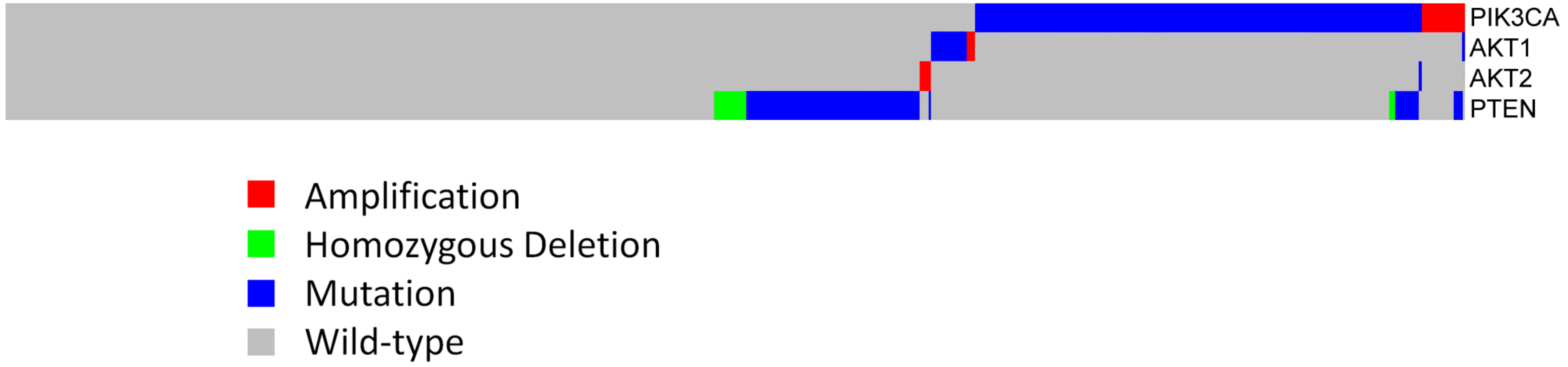

Supplementary figure 4: Mutual exclusivity of PIK3CA with AKT and PTEN.

Mutation status of PIK3CA, AKT1, AKT2 and PTEN in 560 breast cancer cases. Test for mutual exclusivity was performed using the coMEt test: p=3.6e-5.

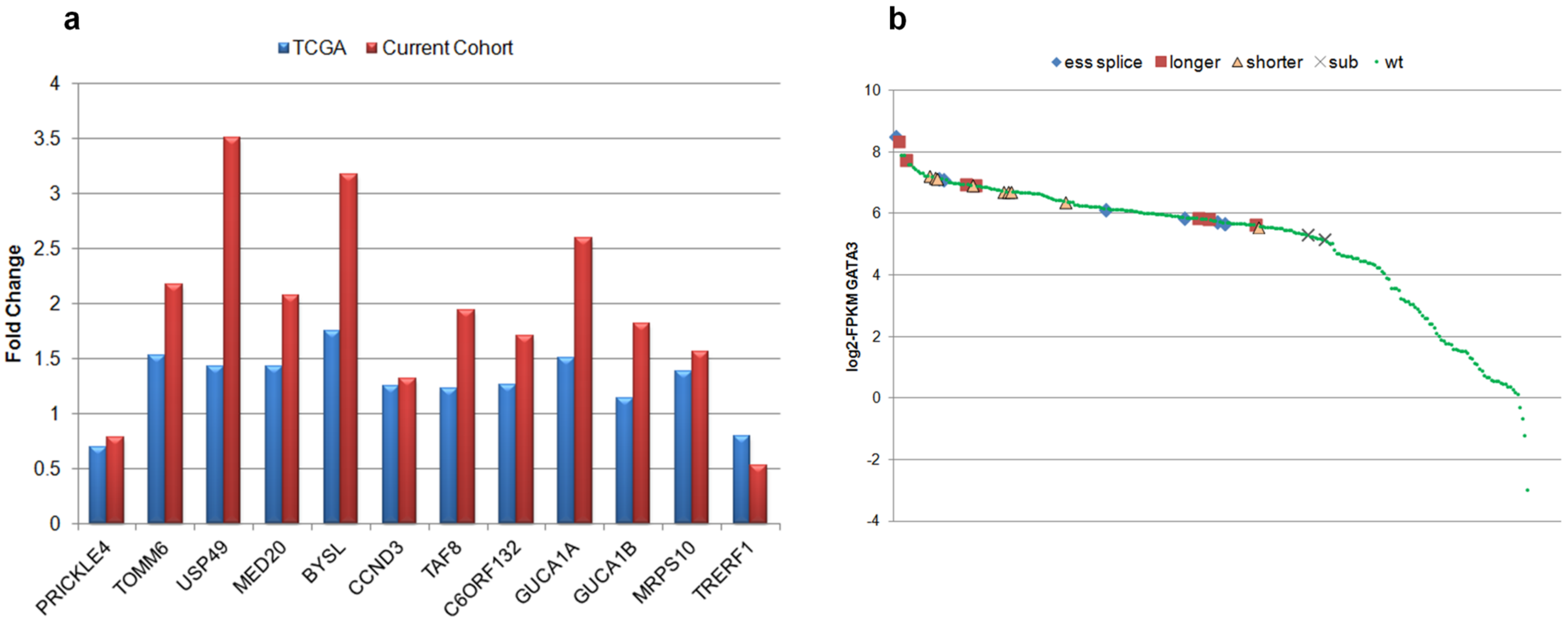

Supplementary figure 5:

A: Fold changes of genes surrounding CCND3. The average expression of genes surrounding CCND3 of cases with and without amplification of the CCND3 locus were used to calculate fold changes. A fold change > 1 indicates genes with a higher average expression of that gene in amplified cases. Genes are ordered by chromosomal location. For TCGA data, samples with a "Gain" and "Amplification" were combined as a single group and compared against the diploid cases.

B: Expression level GATA3 by type of mutation. wt=wild-type, sub=substitution, longer=frameshifts leading to a longer protein than WT, shorter=frameshifts leading to shorter proteins than WT.

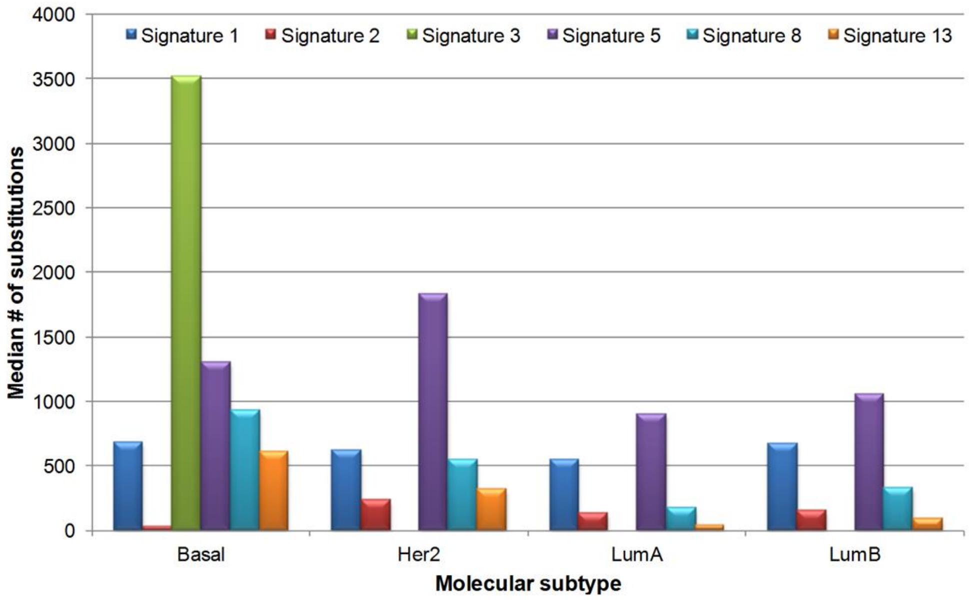

AIMS subtype	n	Sig 1	Sig 2	Sig 3	Sig 5	Sig 6	Sig 8	Sig 13	Sig 17	Sig 18	Sig 20	Sig 26	Sig 30
		median nr of substitutions											
Basal	64	680.5	33.5	3514.5	1305.5	0.0	929.5	607.5	0.0	0.0	0.0	0.0	0.0
Her2	10	626.0	236.5	0.0	1828.5	0.0	554.5	325.0	0.0	0.0	0.0	0.0	0.0
LumA	76	547.5	139.0	0.0	904.5	0.0	180.5	40.0	0.0	0.0	0.0	0.0	0.0
LumB	111	676.0	158.0	0.0	1057.0	0.0	329.0	91.0	0.0	0.0	0.0	0.0	0.0
	Kruskal-Wallis	0.0259	<0.0001	<0.0001	<0.0001	0.7170	<0.0001	<0.0001	0.0172	0.1406	1.0000	0.4380	0.3797

	Contrast	p	p	p	p	NA	p	p	p	NA	NA	NA	NA
Post-hoc analysis*	Basal v Her2	1.0000	0.0033	<0.0001	0.2553		1.0000	1.0000	1.0000				
	Basal v LumA	0.1204	0.0036	<0.0001	0.0003		<0.0001	<0.0001	0.0784				
	Basal v LumB	1.0000	<0.0001	<0.0001	0.0140		0.0577	<0.0001	0.3960				
	Her2 v LumA	1.0000	0.4510	1.0000	0.0003		0.0773	0.0034	0.0991				
	Her2 v LumB	1.0000	0.8925	1.0000	0.0026		1.0000	0.0817	0.2471				
	LumA v LumB	0.0228	1.0000	1.0000	0.8917		0.0083	0.1104	1.0000				

Supplementary figure 6: Number of substitutions by signature versus molecular subtype.

Molecular subtypes were established using the AIMS method. * p-values corrected for multiple testing (Bonferroni). NA: Not Allowed. The Normal-like subtype was excluded from the analysis due to low numbers (n=5).

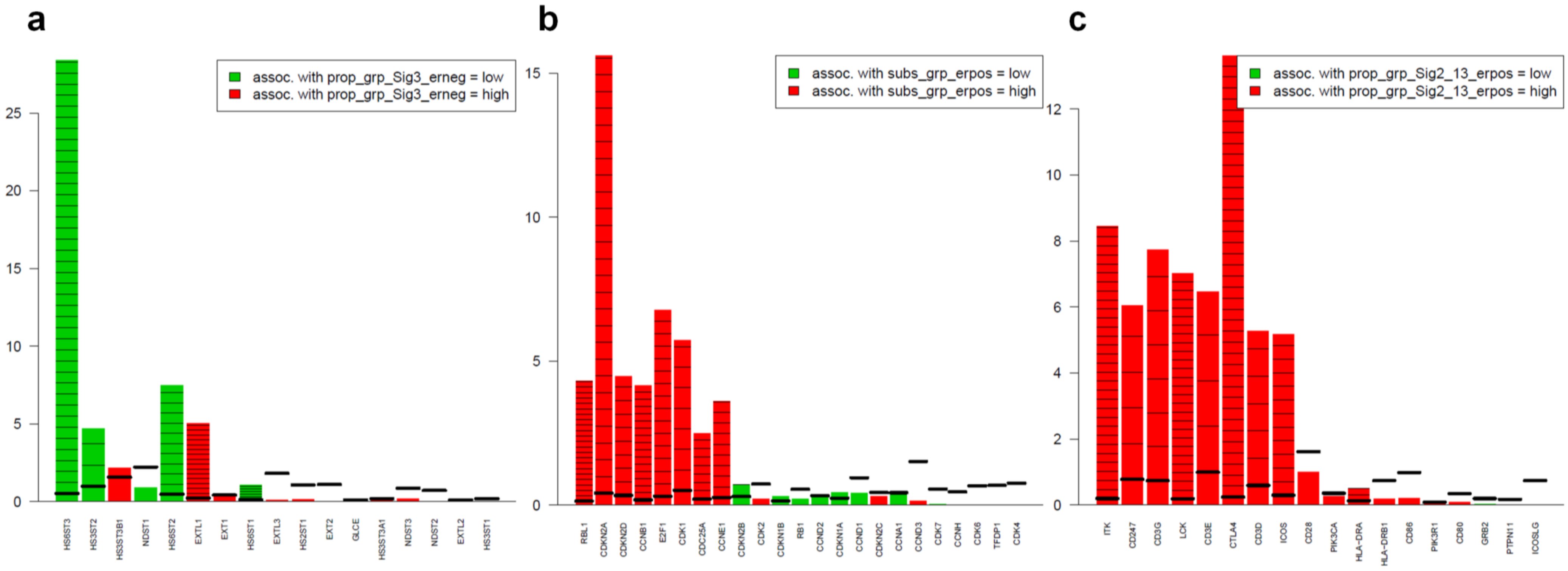

Supplementary figure 7: Geneplots pathway analysis.

a) Heparan sulfate biosynthesis (KEGG) associated in cases with a low proportion of Signature 3. b) Cyclins and Cell Cycle Regulation (Biocarta) associated in cases with a absolute high number of substitutions. c) The CoStimulatory Signal During Tcell Activation (Biocarta) associated in cases with a high proportion of Signature 2+13 (The *CTLA4* gene is the 6th bar). All plots show bars as individual genes in a pathway, the height of the bar is the weighted test statistic. The horizontal thick and thin black bars are the mean and standard deviation under the null hypothesis (see methods for references).

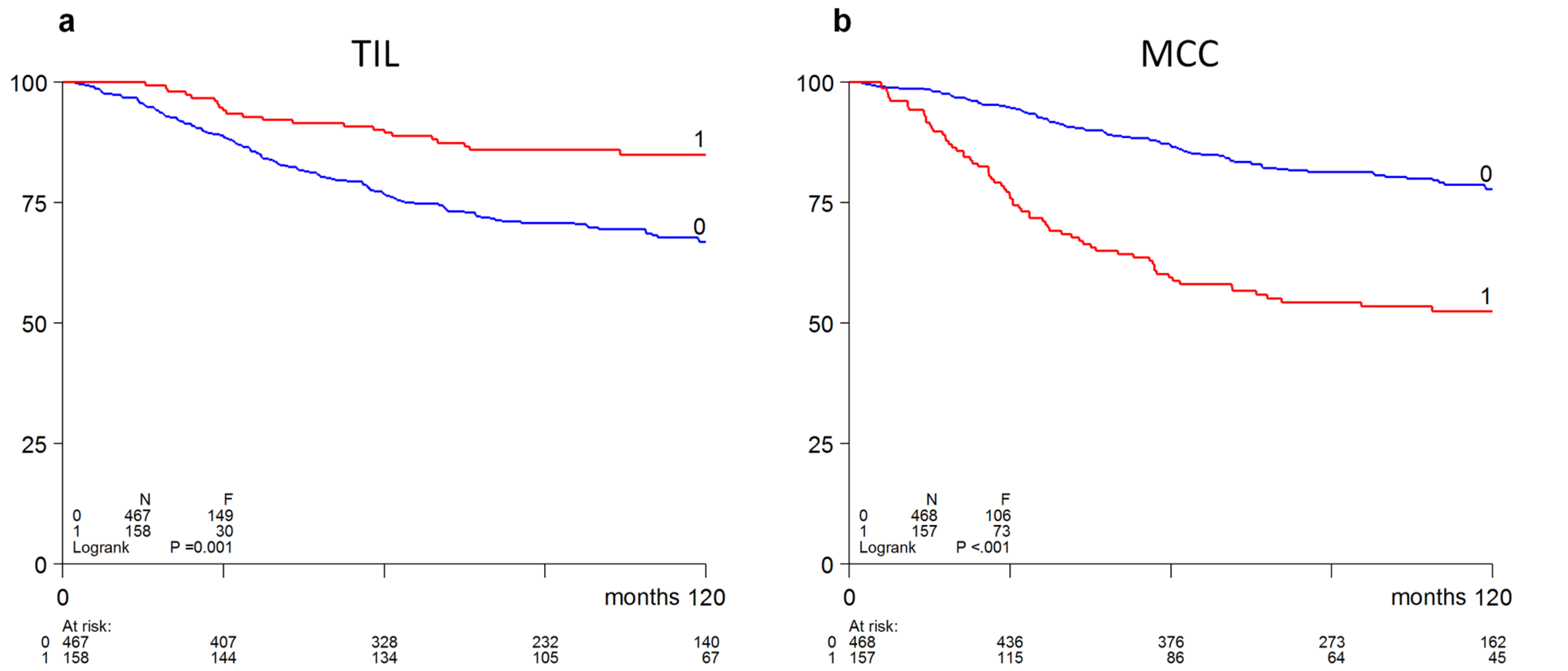

Supplementary figure 8: Metastasis-free survival in 625 ER-positive breast cancer cases.

Red lines (1) indicate patients with high expression (top quartile) of genes of a tumor infiltrating lymphocytes (TIL) signature (**a**) or mitotic cell cycle genes (MCC, **b**). Blue lines (0) indicating patients with low expression (bottom 3 quartiles) of said signatures. P-values are logrank-test values. The x-axis shows time in months, y-axis shows the proportion of patients. Patients at risk at the indicated time points are provided at the bottom.

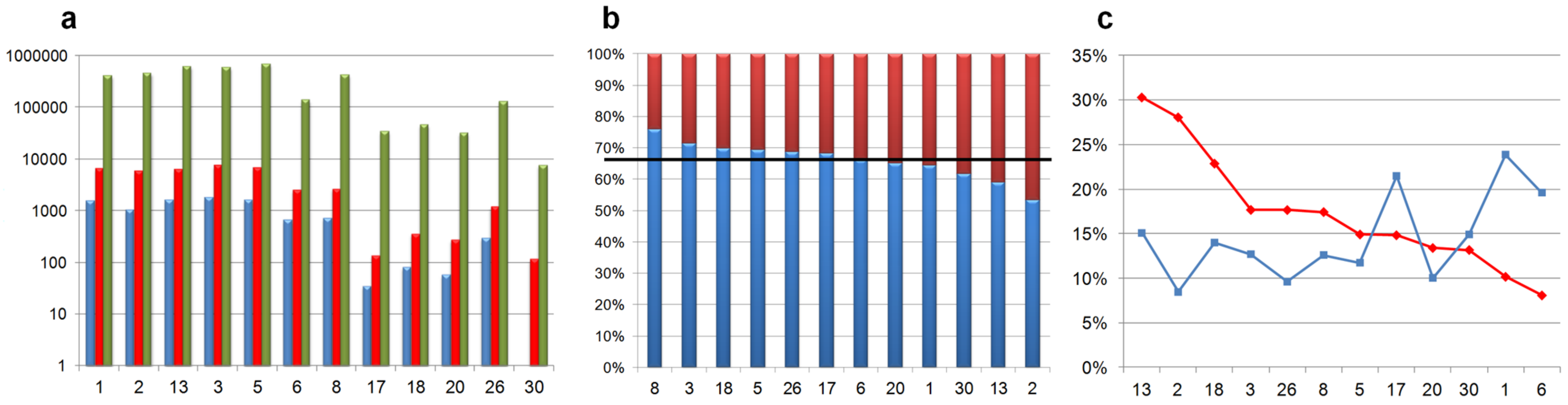

Supplementary figure 9: Amino-acid properties by signature.

X-axis shows the signature number in all three panels. **a:** The number of DNA substitutions of the diverse signatures, grouped by all substitutions (green bar), those substitutions which change amino-acids (red bar) and the substitutions which are predicted as neo-epitopes (blue bar). **b:** The proportion of mutated amino-acids that lead to a change in hydrophobicity (blue bar) or no change (red bar) of the protein. The horizontal black line is the overall change, regardless of signature type. **c:** The proportion of mutated amino-acids that lead to an increase (red line) or decrease in charge (blue line) of the protein.

Database	Name	ERNEG_Sig3	ERPOS-Sig5	ERPOS_AllSubs	ERPOS_Sig2+13
KEGG	Heparan sulfate biosynthesis	0.0033			
	Glycan structures biosynthesis 1	0.0094			
	Glycosphingolipid biosynthesis neolactoseries	0.0174			
	Phenylalanine metabolism	0.0094			
	Olfactory transduction	0.0123			
	Androgen and estrogen metabolism	0.0170			
	Arachidonic acid metabolism	0.0174			
	Urea cycle and metabolism of amino groups	0.0174			
	Keratan sulfate biosynthesis	0.0174			
	Pentose and glucuronate interconversions	0.0268			
	Porphyrin and chlorophyll metabolism	0.0243			
	Tyrosine metabolism	0.0174			
	Arginine and proline metabolism	0.0207			
	Focal adhesion	0.0243			
	Tight junction	0.0174			
	Parkinsons disease		0.0390		
	Glycosaminoglycan degradation		0.0390		
	Alanine and aspartate metabolism		0.0390	0.0102	
	**Cell cycle**		0.0355	0.0024	
	NGlycan degradation		0.0390		
	Glycan structures degradation		0.0390		
	betaAlanine metabolism		0.0355	0.0102	
	Hedgehog signaling pathway		0.0355		
	Glutamate metabolism		0.0390	0.0113	
	DNA polymerase			0.0127	
	Butanoate metabolism			0.0113	
Biocarta	Role of EGF Receptor Transactivation by GPCRs in Cardiac Hypertrophy	0.0195			
	IL7 Signal Transduction				0.0408
	Stathmin and breast cancer resistance to antimicrotubule agents				0.0408
	The CoStimulatory Signal During Tcell Activation				0.0408
	Activation of Csk by cAMPdependent Protein Kinase Inhibits Signaling through the T Cell Receptor				0.0408
	Role of Tob in Tcell activation				0.0408
	NO2dependent IL 12 Pathway in NK cells				0.0408
	IL12 and Stat4 Dependent Signaling Pathway in Th1 Development				0.0408
	T Cell Receptor Signaling Pathway				0.0408
	Caspase Cascade in Apoptosis			0.0339	0.0408
	IL 2 signaling pathway				0.0493
	Selective expression of chemokine receptors during Tcell polarization				0.0493
	IL 17 Signaling Pathway				0.0493
	CTCF First Multivalent Nuclear Factor			0.0361	
	B Cell Survival Pathway			0.0010	
	**Cyclins and Cell Cycle Regulation**			0.0009	
	**CDK Regulation of DNA Replication**			0.0186	
	Tumor Suppressor Arf Inhibits Ribosomal Biogenesis			0.0044	
	METS affect on Macrophage Differentiation			0.0173	
	Estrogenresponsive protein Efp controls cell cycle and breast tumors growth			0.0186	
	**Cell Cycle G1 S Check Point**			0.0009	
	Role of BRCA1 BRCA2 and ATR in Cancer Susceptibility			0.0337	

Supplementary table 1: Pathway analysis results

Bonferroni-Holm corrected p-values, by pathway name. Names in bold relate to cell cycle pathways, in italics are pathways related to immune response.

Signature	n	p-value proportion	p-value absolute numbers	average proportion top quartile	average proportion bottom 3 quartiles	average number top quartile	average number bottom 3 quartiles
1	183	0.0006	0.0005	20.2	27.7	532.8	752.5
2	179	0.62	0.64	13.7	9.6	2313.1	328.0
3	24	0.036	0.027	7.0	3.1	581.4	209.5
5	166	0.19	0.27	32.7	38.3	1033.0	1022.2
6	2	nd	nd	0.5	0	17791.0	0
8	123	0.65	0.59	13.3	12.4	663.6	453.3
13	115	0.0032	0.0027	11.4	6.0	1897.0	309.5
17	6	nd	nd	0.1	0.3	9.7	23.0
18	32	0.35	0.39	0.9	2.0	37.8	64.4
20	1	nd	nd	0	0	0	0
26	3	nd	nd	0.2	0.6	171.5	87.6
30	1	nd	nd	0	0	0	0

Supplementary table 2: Number of substitutions by signature in the TIL-signature groups

P-values are derived from Mann-Whitney ranksum test. Samples were grouped according to the average expression of TIL-signature genes in the top quartile and the bottom 3 quartiles. N indicates the number of patients with > 0 substitutions of the signature. The average proportion or absolute number of substitutions of a signature was calculated.

Signature	N	p-value proportion	p-value absolute numbers	average proportion Nill	average proportion Mild	average proportion Moderate / Severe	average number Nill	average number Mild	average number Moderate / Severe
1	253	0.015	0.537	25.8	26.7	19.3	592.9	728.5	649.9
2	248	0.308	0.022	6.8	10.4	11.0	169.5	657.7	1401.5
3	36	<0.0001	<0.0001	2.3	2.7	12.6	127.7	197.8	944.2
5	237	<0.0001	0.158	47.6	37.6	27.7	1131.2	1040.7	1059.4
6	2	nd	nd	0	0.2	0.5	0	54.9	355.8
8	172	0.343	0.117	12.9	11.9	14.5	322.4	435.0	785.4
13	174	<0.0001	<0.0001	3.1	7.0	13.1	91.5	573.1	1613.9
17	9	nd	nd	0.2	0.3	0.2	11.7	20.9	14.5
18	45	0.905	0.935	1.3	2.4	0.9	32.6	90.3	34.2
20	0	nd	nd	0	0	0	0	0	0
26	3	nd	nd	0	0.7	0.2	0	126.0	164.6
30	0	nd	nd	0	0	0	0	0	0

Supplementary table 3: Number of substitutions by signature in the lymphocytic infiltrate groups

P-values are derived from p-value of Cuzick's nonparametric test for trend across ordered groups. Samples were grouped according to the pathological lymphocytic infiltrate status. N indicates the number of patients with > 0 substitutions of the signature. The average proportion or absolute number of substitutions of a signature was calculated per group.
